# The Link between Different Types of Prebiotics in Infant Formula and Infection Rates: A Review

**DOI:** 10.3390/nu15081942

**Published:** 2023-04-18

**Authors:** Roxane Cool, Yvan Vandenplas

**Affiliations:** Vrije Universiteit Brussel (VUB), UZ Brussel, KidZ Health Castle, 1090 Brussels, Belgium

**Keywords:** prebiotic, fructooligosaccharide, galactooligosaccharide, inulin, human milk oligosaccharide, infant formula, infection, antibiotic

## Abstract

Breastfeeding plays a protective role against infections, partially through the prebiotic effect of human milk oligosaccharides (HMOs). Aiming to mimic these beneficial capacities, there is an ongoing search to make infant formula closer to human milk, including by adding oligosaccharides. Over the past two decades, multiple studies have been published on different types of prebiotics and their role in reducing infection rates in infants. This review aims to answer the question of whether there is evidence that the addition of oligosaccharides to infant formula decreases the prevalence of infection, and whether the effect is influenced by the kind of oligosaccharide added. The review of the literature reveals an important heterogeneity, including different types and dosages of prebiotics, different intervention periods and inclusion criteria, etc., making it impossible to formulate a consensus about the efficacy of adding prebiotics to infant formula. We would cautiously suggest that supplementation with galactooligosaccharides (GOSs)/fructooligosaccharides (FOSs) seems to have a beneficial effect on infection rates. For HMOs, more studies about the different types of HMOs are necessary to make any deductions. GOSs alone, inulin, and MOSs (bovine-milk-derived oligosaccharides) do not reduce the incidence of infections. The combination of GOSs and PDX (polydextrose) was found to play a protective role in one study. The evidence of the effect of prebiotics in reducing the use of antibiotics is low. The many lacunas in the direction of study uniformity offer many opportunities for further research.

## 1. Introduction

Human milk offers multiple advantages in comparison to infant formula, such as a more balanced development of the immune system, decreased prevalence of infections, and better cognitive development [1,2,3]. These differences are at least partially related to the differences in the intestinal microbiota composition of breastfed infants compared to formula-fed infants, particularly with respect to *Bifidobacteria* species [4,5,6].

Breast milk is rich in bioactive elements with a protective role against infections, including immunoglobulins, lactoperoxidase, lysozymes, lactobacilli, and human milk oligosaccharides (HMOs) [7]. As the third most abundant component in human milk, HMOs reach concentrations of >20 g/L in colostrum and 12–14 g/L in mature milk during early lactation. There are many confounding variables influencing the amount of HMOs in relation to the duration of lactation [8]. HMOs are complex carbohydrates of which more than 200 types have been identified in human milk. In secretor mothers, 2′-fucosyllactose (2′-FL) is the most common. Only one to a few percent of the HMOs are absorbed through the intestinal wall; the majority of the HMOs reach the colon intact. HMOs are prebiotics with obvious bifidogenic effects on the gut microbiota. Different mechanisms of action have been identified for HMOs, such as anti-adhesive and antimicrobial effects [9,10], decreased intestinal permeability, and modulation of the intestinal immune response [11], infectious diseases [10,12], and immune [13,14] and brain development [15,16]. Some studies tend to link the prebiotic effect with the composition of the HMOs [12].

The mean annual infection rate in children under three years is three to five [17,18] which generates multiple healthcare visits, important morbidity and mortality, but also economic burdens for the family and society [19]. Taking into account the positive effects of breastfeeding on the infection rates, there is an ongoing search to make infant formula closer to mother’s milk. Several tracks are being explored, such as adding probiotics [20], supplementation with DHA/ARA/EPA (docosahexaenoic acid/arachidonic acid/eicosapentaenoic acid) [21], and milk fat globule membrane (MFGM) [22,23].

Another widely investigated track is the supplementation of infant formula with oligosaccharides (OSs) in order to simulate the same advantageous effect as the HMOs. It was initially impossible to synthesise HMOs; thus, the first trials used OSs such as fructo- or galactooligosaccharides (FOSs, GOSs), inulin, polydextrose (PDX), etc., It was only after 2014 that it became possible to synthesise OSs in sufficient amounts and at sufficient quality with an identical structure to that of HMOs through precision fermentation by genetically modified microorganisms. There are currently only a limited number of HMOs available and approved for use in infant formula and in nutritional supplements. These HMOs include 2′-FL, 3′-FL, DFL, LNT, LNnT, 3′-SL, and 6′-SL. These synthetic HMOs are all short-chain OSs.

Multiple randomised controlled trials (RCTs) were performed during the past two decades to improve our understanding of the potential beneficial effects of prebiotic OSs added to infant formula. All of these studies showed that the currently established prebiotic OSs are well tolerated [24,25]. Some studies tend to show a reduction in the prevalence of infections and antibiotic prescriptions [26,27,28]. However, these results are disputed in other studies reporting no benefit. Systematic reviews on the effect of prebiotics on infection could no show a difference according to the type of prebiotic [24,29]. It remains unclear whether these outcomes differ for synthesised human OSs or other established prebiotic OSs. This article aims to review the literature regarding the effects of different types of prebiotics on infection rates and antibiotic use.

## 2. Materials and Methods

This review aimed to answer the question of whether there is evidence that the addition of oligosaccharides to infant formula can decrease the prevalence of infection, and whether the effect is influenced by the kind of oligosaccharide added (Table 1). 

To answer this question, a literature search was conducted in PubMed and Embase. The literature search strategy was formulated using the following terms:

Oligosaccharide OR FOS OR GOS OR fructooligosaccharide OR fructo-oligosaccharide OR galactooligosaccharide OR galacto-oligosaccharide OR prebiotic OR HMO OR human milk oligosaccharide OR LNnT OR 2′FL OR inulin OR PDX OR polydextrose.
AND infection OR infectious OR respiratory tract infection OR RTI OR URTI OR fever OR antibiotic. AND formula milk OR milk OR formula OR infant formula.

We also cross-checked references cited in RCTs and (systematic) reviews on the subject. We only included RCTs published in English. There were no restrictions on the date of publication or on the duration of the trials. There were no exclusion criteria regarding the type of infant formula or gender. The focus of this review is on prebiotics. Depending on how they were used, studies with complementation of probiotics were included. If the probiotics were only added in the intervention group, the study was excluded. Studies where probiotics were administered to both the intervention and the control group were included. In case of lack of clarity in the article, an email was sent to the designated corresponding author. In case of persistent ambiguity, the article was excluded.

## 3. Results

After our literature search, we selected 17 publications, which we divided into three groups. 

### 3.1. Galactooligosaccharides (GOSs)/Fructooligosaccharides (FOSs)

We found six publications reporting on four trials with short-chain (sc) GOSs and long-chain (lc) FOSs, as listed in Table 2 [26,27,30,31,32,33]. All patients were healthy term infants born in Europe. Two trials reported short- and long-term observations (six months [30] to two years [26] and one year [32] to five years [33]). A total of 2206 participants were included, of whom 946 participants received scGOSs/lcFOSs in a range of 4 to 8 g/L. A total of 960 participants were included in the control group and received unsupplemented infant formula. In the study of van Stuijvenberg et al. [32,33], a third group with breastfeeding was included as a reference group. One study was not blinded [27]. In one study, only patients with a parental history of atopy were included [26,30], whereas parental atopic history was an exclusion criterion in two other studies [31,32,33]. 

In the trial reported by Arslanoglu et al., the effect of 8 g/L GOSs/FOSs was evaluated in infants with a parental atopic history. During the six months of intervention, a difference was noted in the prevalence of infections (*p* = 0.01), recurrent infections (*p* < 0.05), and recurrent upper respiratory tract infections (*p* < 0.05) [30]. Antibiotic intake tended to be lower, but this was not statistically significant (*p* = 0.1). After an observation of two years, the number of infections was still lower (*p* < 0.01), and a statistically significant difference in antibiotic use was observed (*p* < 0.05) [26]. There were also less upper respiratory tract infections (URTIs) (*p* < 0.01). Furthermore, this trial showed a trend of a lower incidence of urinary tract infections (*p* = 0.06).

In an open controlled trial by Bruzzese et al., infants were given 4 g/L GOSs/FOSs over the course of one year [27]. The included patients were 15 to 120 days old at inclusion and had a minimum of two weeks of exclusive breastfeeding. In this study, the prevalence of diarrhoea episodes was lower (*p* = 0.015). The number of URTIs was comparable (*p* = 0.4), but the number of recurrent URTIs tended to be lower (*p* = 0.06). Only 60% of the participants had at least one URTI, but there were no precise observations about the annual infection rate per child. The mean antibiotic use was lower (*p* = 0.038), as was the number children receiving two or more antibiotic courses per year (*p* = 0.004). 

**Table 2:** Overview of references with GOSs/FOSs.

First Author, Year [Reference]	Country	Inclusion Criteria; Age at Inclusion	Intervention (Number Included)	Control (Number Included)	Duration of a. Intervention b. Observation	Outcomes
**Arslanoglu,** **2007** [30]	Italy	Term infants with parental atopic history; <2 weeks	EHF + GOSs/FOSs 9:1 8 g/L (+/− BF first 6 weeks) (102)	EHF (104)	a. 6 months b. 6 months	Primary outcomes ● Infectious episodes: 21/102 vs. 47/104 (*p*= 0.01) ● Infections requiring antibiotics: 11/102 vs. 22/104 (*p*= 0.1) ● Recurrent infections: 4/102 vs. 13/104 (*p* < 0.05) ● URTIs: 14/102 vs. 30/104 (*p*=0.07) ● GI infections: 1/102 vs. 4/104(*p* =0.18) Secondary outcome ● Faecal *Bifidobacteria*: mean 10.3 vs. 8.7 colony-forming units/g stool (*p* < 0.01)
**Arslanoglu,** **2008** [26]	Italy	Term infants with parental atopic history; <2 weeks	EHF + GOSs/FOSs 9:1 8 g/L (+/− BF first 6 weeks) (66)	EHF (68)	a. 6 months b. 2 years	Primary outcomes ● Allergic manifestations (*p* < 0.05) - Atopic dermatitis 13.6% vs. 27.9% - Recurrent wheezing 7.6% vs. 20.6% - Allergic urticaria 1.5% vs. 10.3% Secondary outcomes ● Growth: mean body weight and length were similar ● Infectious episodes - Overall infections: mean 4.1 ± 3.1 vs. 5.9 ± 4.1(*p* < 0.01) - URTIs: mean 2.1 ± 1.8 vs. 3.2 ± 2.2 (*p* < 0.01) - LRTIs: mean 0.9 ± 1.1 vs. 1.3 ± 0.8 (*p* > 0.05) - GI infections: mean 0.4 ± 0.7 vs. 0.6 ± 0.9(*p* > 0.05) - Infections requiring antibiotics: mean 1.8 ± 2.3 vs. 2.7 ± 2.4 (*p* < 0.05) - Urinary tract infections: 0 ± 0 vs. 0.1 ± 0.5 (*p* = 0.06)
**Bruzzese,** **2009** [27]	Italy	Healthy term infants after at least 2 weeks of exclusive breastfeeding; 15–120 days	Infant formula + GOSs/FOSs 9:1 4 g/L (169)	Infant formula (173)	a. 1 year b. 1 year	Primary outcomes ● Diarrhea episode/child: mean 0.12 ± 0.04 vs. 0.29 ± 0.05 (*p* = 0.015) ● URTIs: 60/94 vs. 65/109 (*p* = 0.4) ● Recurrent URTIs: 17/60 vs. 29/65 (*p* = 0.06) ● LRTIs: 4/94 vs. 6/109 (*p* > 0.05) ● Antibiotics prescribed: 1.03 ± 0.15 vs. 1.48 ± 0.16 (*p* = 0.038) Secondary outcomes ● Growth - Average weight, length, and head circumference: similar in both groups - Mean body weight: increased at 3 and 6 months, similar at 9 and 12 months - Mean body length: greater (*p* < 0.05) - Mean head circumference: similar
**Bocquet,** **2013** [31]	France	Healthy term infants; <42 days	Infant formula + *B. lactis* + GOSs/FOSs 9:1 4 g/L (261)	Infant formula + *B. lactis* (267)	a. 1 year b. 1 year	Primary outcomes ● Mean number of infections: 4.9 ± 3.2 vs. 4.5 ± 3.2 (*p* = 0.18) ● No differences in type of infection - GI infections (*p* > 0.1) - Antibiotic use: 1.0 ± 1.2 vs. 0.9 ± 1.2 (*p* = 0.3) Secondary outcomes ● Anthropometric measurements: similar in both groups ● Tolerance: no differences in daily stool frequency and consistency or overall acceptance of the formula ● Adverse events: 60% in each group had one or more AE; none was related to the study formula
**van Stuijvenberg,** **2011** [32]	Netherlands, Austria, Switzerland, Italy, Germany	Healthy term infants; <8 weeks	Infant formula + GOSs/FOSs 9:1 6.8 g/L + pectine-derived acidic OSs 1.2 g/L (414)	● Infant formula (416) ● Breastfeeding (300)	a. 1 year b. 1 year	Primary outcomes ● Fever episodes: - ITT analysis: median 1.19 [0.2–9.34] vs. 1.16 [0.2–6.38] (*p* > 0.05) - PP analysis for first 6 months: median 0.13 [0.12–2.31] vs. 0.13 [0.12–2.36] (*p* < 0.05) ● Antibiotic use: median 0.05 [0.05–0.11] vs. 0.05 [0.05–0.16] (*p* > 0.05)
**van Stuijvenberg,** **2015** [33]	Netherlands, Austria, Switzerland, Italy, Germany	Healthy term infants; <8 weeks	Infant formula + GOSs/FOSs 9:1 6.8 g/L + pectine-derived acidic OSs 1.2 g/L (232)	● Infant formula (243) ● Breastfeeding (197)	a. 1 year b. 5 years	Primary outcomes ● Fever episodes at 3 to 5 years: median episodes per year 1.17 [0.5–2.08] vs. 1.2 [0.52–2.57] (*p* = 0.22) ● Episodes of coughing, wheezing, vomiting, and diarrhoea: similar (*p* > 0.1) Secondary outcomes ● Duration of diarrhoea in days: median 1 (0–4) vs. 2 (0–7) (*p* = 0.01) ● Duration of coughing, wheezing, rhinitis, and vomiting: similar (*p* > 0.1) ● Runny nose: median episodes per year in the breastfed infants 3.62 [1.97–5.76] vs. intervention group 2.59 [1.02–5.26] vs. control group 2.19 [0.99–4.95] ● Antibiotic use: 172/232 vs. 203/243 (*p* > 0.05) ● Antipyretic use: 224/232 vs. 237/243 (*p* > 0.05)

Results are written as absolute numbers, as mean values ± standard deviations, or as median values [25–75th percentiles]. EHF: extensively hydrolysed formula, BF: breastfeeding, GOS: galactooligosaccharide, FOS: fructooligosaccharide, OS: oligosaccharide, U/L RTI: upper/lower respiratory tract infection, GI: gastrointestinal, ITT: intention to treat, PP: per protocol.

Bocquet et al. investigated the effects of 4 g/L GOSs/FOSs added to infant formula [31]. *Bifidobacterium animalis* subspecies *lactis* (*B. lactis*) was added to both the control and intervention formula, given to 278 and 290 infants, respectively. After an intervention and observation time of 12 months, there were no differences in the overall infection rates (*p* = 0.18), nor in the different types of infections (i.e., upper and lower RTIs, gastrointestinal infections, and others). There were no differences in antibiotic use (*p* = 0.3).

The fourth study explored the outcomes of 6.8 g/L GOSs/FOSs and 1.2 g/L pectine-derived acidic OSs added to infant formula (*n* = 414), compared to standard formula (*n* = 416) and breastfeeding (*n* = 300), during an intervention period of 12 months. The primary outcome was the number of episodes of fever, defined by a rectal temperature > 38.5 °C. After the observation periods of 1 and 5 years, no differences in the occurrence of fever episodes were reported (*p* > 0.05 [32] and *p* = 0.22 [33], respectively). However, in a per protocol analysis, a reduction in fever in the first 6 months was reported (*p* < 0.05) [32]. Overall, a low infection rate (1.15/child/year) was reported. However, it is noteworthy that more infection episodes were reported in the breastfeeding group, especially for ‘runny or blocked nose’.

### 3.2. Human Milk Oligosaccharides

We retained six publications on human milk oligosaccharides (HMOs) (Table 3) [28,34,35,36,37,38] involving a total of 1571 infants, conducted in America, Europe, and Asia. Five studies included only healthy term infants, and one study examined the effects of HMOs on infants with cow’s milk allergy (CMA). HMOs were added in a range of 0.25 g/L to 5.8 g/L, using 2′-FL, LNnT, 3′-FL, LNT, 3′-SL, and 6′-SL.

The first study was published in 2015 by Marriage et al. In this study, four groups were compared: (i) formula supplemented with 2.2 g/L GOSs and 0.2 g/L 2′-FL; (ii) 1.4 g/L GOSs and 1 g/L 2′-FL; (iii) in the control group, infant formula with 2.4 g/L GOSs; and (iv) a breastfed reference group. After an intervention and observation period of four months, there were more infections in the second intervention group (38/109) and in the control group (28/109) compared to the first intervention group (11/104) (*p* < 0.05) [34]. These results were not compared to the breastfeeding group. It should be noted that the control group received a prebiotic as well, and that the total dosage of prebiotic was 2.4 g/L in each group. Considering this study setup and its results, which are difficult to interpret, this study was not considered in our conclusions.

In the study by Puccio et al., formula was supplemented with 2′-FL and LNnT for six months [28]. After an observation period of 12 months, fewer infections (*p* = 0.051) and a decreased use of antibiotics (*p* < 0.05) were reported. Furthermore, there was less bronchitis (*p* < 0.01) and a lower incidence of RTIs (*p* < 0.05). 

Storm and co-workers added a small amount of 2′-FL (0.25 g/L) to partially hydrolysed formula containing *B. lactis* over a period of six weeks. The control formula contained *B. lactis* as well. In the adverse events, they observed a reduction in the incidence of infections, which was low in both groups (9/38 vs. 3/40; *p* = 0.05) [35]. Due to the small sample of participants (78 infants), short period of intervention, and low infection rate, this study was not included in our conclusions.

In two RCTs, published by Parschat and Lasekan, a mix of five HMOs (5.75 g/L) was added to infant formula over the course of four months. Both investigations reported no decrease in infection rates. In the study of Parschat et al., the infection rate was 32/113 vs. 28/112 (*p* = 0.54) [36]. In the study of Lasekan et al., the antipyretic use was comparable (4/128 and 4/126), as was the antibiotic use (4/128 vs. 4/126) [38]. However, the study of Lasekan was conducted during the COVID-19 pandemic, where the infection rates in infants were generally low (7.6% of included patients), which is likely to have had an impact on these observations. 

**Table 3:** Overview of references with HMOs.

First Author, Year [Reference]	Country	Inclusion Criteria; Age at Inclusion	Intervention (Number Included)	Control (Number Included)	Duration a. Intervention b. Observation	Outcomes
Marriage, 2015 [34]	USA	Healthy term infants; <5 days	● Test 1 (T1) Infant formula + GOSs 2.2 g/L + 2′-FL 0.2 g/L (104) ● Test 2 (T2) Infant formula + GOSs 1.4 g/L + 2′ -FL 1 g/L (109)	● Control (C) Infant formula + GOSs 2.4 g/L (101) ● Breastfeeding (BF) (106)	a. 4 months b. 4 months	Primary outcome Anthropometric measures: no difference in mean weight, length, and head circumference Secondary outcomes ● Mean daily formula intake: similar ● Tolerance: - Stool per day: more in BF group, similar in T1–T2–C group (*p* < 0.01) - Stool consistency: higher in formula groups vs. BF - Spitting and vomiting: more in T1–T2–C group versus BF group (*p* < 0.05) ● Adverse events: - More infections in C and T2 groups (RTI, OM, viral infections, oral candidiasis): Test 1 11/104 vs. Test 2 38/109 vs. Control 28/101 (*p* < 0.05) - Eczema: more in C group (5) than T1 and T2 (0) (*p* < 0.05) ● 2′FL uptake in plasma and excretion in urine: - Plasma uptake higher in BF > T2 > T1 - Urine excretion BF, T2 > T1 > C
Puccio, 2017 [28]	Italy, Belgium	Healthy term infants; <14 days	Infant formula + 2′-FL 1 g/L + LNnT 0.5 g/L (88)	Infant formula (87)	a. 6 months b. 12 months	Primary outcome Weight gain: similar in both groups Secondary outcomes ● Other anthropometric measures: similar ● Formula intake: similar ● GI tolerance: similar ● Stools softer at 1 and 2 months, no differences > 2 months ● Behavioural patterns: similar ● Morbidity - Infants with at least 1 infection: 69.3% vs. 82.8% (*p* = 0.051) - Bronchitis: 9/88 vs. 24/87 (*p* < 0.01) - LRTIs: 17/88 vs. 30/87 (*p* < 0.05) - Antibiotic use: 37/88 vs. 53/87 (*p* < 0.05)
Storm, 2019 [35]	USA	Healthy term infants; <19 days	Partially hydrolysed formula + *B. lactis*+ 2′ -FL 0.25 g/L (38)	Partially hydrolysed formula+ *B. lactis* (40)	a. 6 weeks b 6 weeks	Primary outcome Tolerance: GSQ 20.9 ± 4.8 vs. 20.7 ± 4.3 (*p* = 0.82) Secondary outcomes ● Stool frequency, consistency, and ease of passing: similar ● Spit up, vomiting, crying, fussing: no difference ● Formula intake: similar ● Adverse events: - infections: 9/40 vs. 3/38—*p* 0.05-URTI: 0/38 vs. 4/10 (*p* = 0.12) ● Anthropometric measures: similar
Parschat, 2021 [36]	Germany, Italy, Spain	Healthy term infants; <14 days	Infant formula + 2′ FL 2.99 g/L + 3 FL 0.75 g/L + LNT 1.5 g/L + 3′ SL 0.23 g/L + 6′ SL 0.28 g/L (113)	● Infant formula (112) ● Breastfeeding (116)	a. 4 months b. 6 months	Primary outcome Weight gain: none inferior Secondary outcomes ● Other anthropometric measures: similar ● GI tolerance: similar ● Defaecation: stools softer and more frequent ● Adverse events: - Incidence of AEs was similar in CG and TG, higher than the BF group - Incidence of infections similar in the 3 groups: 32/113 vs. 28/112 vs. 34/116 (*p* = 0.5 and 0.8)
Vandenplas, 2022 [37]	Europe, Singapore	Infants with CMPA; <6 months	EHF + 2′ FL 1 g/L + LNnT 0.5 g/L Reduced protein content (2.2 g/dL) (94)	EHF (protein 2.47 g/dL) (96)	a. 4 months b. 4 months	Primary outcome Weight gain: none inferior Secondary outcomes ● Other anthropometric measures: similar ● Safety:rate of AE similar ● Morbidity: infections - LRTIs: 13/94 vs. 20/6 (*p* = 0.25); RRR 34% - URTIs: 60 in 39 infants (41.5%) vs. 94 in 42 infants (43.8%) (*p* = 0.77); RRR 5.2% - Frequency URTIs: 1.5 vs. 2.2 episodes/year; 0.09 vs. 0.15 episode/month ( *p* = 0.003) - GI infections: 10/94 vs. 17/96 (*p* = 0.21);—RRR 40% - Other viral infections: 19/94 vs. 19/96(*p* =1) - Urinary tract infections: 4/94 vs. 0/96 (*p* = 0.06) - Antibiotic use: 23/94 vs. 25/96 (*p* = 0.82) - Antipyretic use: 35/94 vs. 40/96 (*p* = 0.6)
Lasekan, 2022 [38]	USA	Healthy term infants; <14 days	Infant formula+ 2′ FL 3 g/L+ 3 FL 0.8 g/L+ LNT 1.5 g/L+ 3′ SL 0.2 g/L+ 6′ SL 0.3 g/L (130)	● Infant formula (129) ● Breastfeeding (104)	a. 4 months b. 4 months	Primary outcome Weight gain and other anthropometric measures: similar Secondary outcomes ● GI tolerance - Vomit/spit up: similar (*p* = 0.5) - Stools softer (higher MRSC (*p* = 0.038) and more frequent (*p* = 0.004) ● Adverse events: - Number of AEs similar: 31.7% vs. 32% vs. 26.5% - Antipyretic use: 4/130 vs. 4/129 vs. 8/104 (*p* > 0.05) - Antibiotic use: 4/130 vs. 4/129 vs. 4/104 (*p* > 0.05)

Legend: Results are written as absolute values or as mean values ± standard deviations; BF: breastfeeding, CMPA: cow’s milk protein allergy, AE: adverse event, T: test, TG: test group, C: control, CG: control group, GI: gastrointestinal, U/L RTI: upper/lower respiratory tract infection, GOS: galactooligosaccharide, IGSQ: Infant Gastrointestinal Symptom Questionnaire, RRR: relative risk reduction, MRSC: mean rank stool consistency.

When added to extensively hydrolysed formula in a study population of 190 infants, 2′-FL and LNnT were associated with a reduction in the frequency of a range of infections [37]. In the subgroups, there was a risk reduction of 34% for lower RTIs (13/94 vs. 20/96; *p* = 0.25). There was no difference in the incidence of URTIs (*p* = 0.77); however, the frequency of URTIs per year was lower (*p* = 0.003). Further analyses showed a reduction in otitis media (*p* = 0.17; relative risk reduction 70%) and fewer gastrointestinal infections (*p* = 0.021). There were no reductions in antipyretic (*p* = 0.6) or antibiotic use (*p* = 0.8). 

### 3.3. Other Prebiotics

Studies concerning other prebiotics are listed in Table 4. 

When only adding 4.4–5 g/L GOSs to infant formula until the age of one year, no significant reduction in the prevalence of infections was reported [39]. This conclusion was held after observing 365 infants until the age of one year, where there was no reduction in URTIs (*p* = 0.44), recurrent URTIs (*p* = 0.87), episodes of diarrhoea (*p* = 0.36), or in the prescription rate of antibiotics (*p* = 0.48). In this trial, only infections diagnosed by a paediatrician were included.

The combination of high amounts (20–25%) of beta-palmitic acid and 5 g/L GOSs during a period of three to six months did not result in a reduction in overall infections (*p* = 0.63), nor in the gastrointestinal (*p* = 0.75) or respiratory infections (*p* 0.63) [41]. The overall infection rate in this trial was remarkably low (3/22 vs. 2/19).

A publication from 2018 explored the combination of GOSs and PDX supplemented to infant formula given to 400 randomised participants over the course of 48 weeks [40]. The results showed a reduction in RTIs during the 48 weeks (*p* = 0.02), but this difference was no longer detected at the age of 96 weeks (*p* > 0.05). Recurrent RTIs remained lower in the intervention group at 46 and 96 weeks (*p* = 0.04). There was no difference in the total antibiotic use (58/118 vs. 58/104), but there were fewer participants using antibiotics more than three times during the 96 weeks (*p* = 0.05). There were no statistically significant differences in the number of episodes of acute diarrhoea (62/118 vs. 65/104; *p* > 0.05).

In addition to GOSs, other prebiotics were explored. After a supplementation of 7.2 g/L bovine-milk-derived oligosaccharide (MOS) until the age of six months, Estorminos et al. observed no differences in upper (59/114 vs. 57/112) and lower (24/114 vs. 24/112) RTIs [42]. 

The use of 8 g/L long- and short-chain oligofructose-enriched inulin as a prebiotic added to infant formula for one year did not result in significantly different numbers of infections compared to a standard formula (51 vs. 62%, respectively; *p* = 0.186) [43]. However, enrichment of infant formula with oligofructose-inulin reduced the duration of the infections (*p* = 0.03). The infection rate in infants was generally low in this trial.

## 4. Discussion

### 4.1. Overall Incidence of Infections

When analysing the evidence of the supplementation of infant formula with GOSs/FOSs, half of the studies showed a reduction in the infection rate. The other half showed limited or no infection reduction.

The evidence for a reduction in infection when supplementing infant formula with HMOs is not conclusive. When not including the studies of Storm et al. and Marriage et al., we can conclude that Parschat et al. and Lasekan et al. reported no reduction in infection rates [36,38]. Vandenplas et al. showed a relative risk reduction for infection, but it was not statistically significant [37]. Only Puccio et al. reported a significant reduction in infections, as a secondary outcome [28].

GOSs, alone or supplemented with high-dose β-palmitic acid, did not reduce the incidence of infection [39,41]. The same conclusion was noted for the supplementation of inulin [43] or MOS [42] as prebiotics in infant formula. In one study where GOSs and PDX were combined as a supplement, there was a reduction in RTIs during the period of intervention and a prolonged reduction in recurrent RTIs after ending the prebiotic [40].

### 4.2. Upper Respiratory Tract Infections

Out of four studies, one showed that supplementation with GOSs/FOSs resulted in a reduction in the incidence of URTIs in a short- and long-term observation [26,30]. Another trial did not show a reduction [27]. The other manuscript discussing GOSs/FOSs did not present data on URTIs.

Two studies with HMOs showed no reduction in the total number of episodes of URTIs [28,37], although a reduction in the annual frequency was reported in the second study [37]. The effect on URTIs was not reported in the other trials concerning HMO supplementation.

GOS/PDX supplementation during the first 48 weeks of life was reported to reduce URTIs during this period, but this effect could not be confirmed after 96 weeks [40]. GOSs [39], GOSs with high amounts β-palmitic acid [41], and MOSs [42] did not have an impact on this type of infection.

### 4.3. Lower Respiratory Tract Infection

In all of the trials, the incidence of LRTIs was low or not examined. Focusing on GOSs/FOSs, no reduction in LRTIs was proven in two studies [26,27]. HMOs were associated with a reduction in the incidence of LRTIs in one study [28]. A second trial showed a relative risk reduction of 33.7% after adding HMOs [37]. MOSs had no influence on LRTIs [42]. Other prebiotic studies did not examine LRTIs.

### 4.4. Gastrointestinal Infections

According to data from four studies, GOSs/FOSs do not reduce the rate of gastrointestinal infections [26,30,31,33]. However, in the study of van Stuijvenberg et al. in 2015, a reduction in the duration of infection was reported [33]. In only one study a reduction in GI infections was reported for GOSs/FOSs [27]. Gastrointestinal infections were reported to be statistically insignificantly reduced (*p* = 0.2), with a relative risk reduction of 40% in one HMO study [37]. Supplementation with GOSs [39], GOSs/PDX [40], or βPA/GOSs [41] did not influence this type of infection.

### 4.5. Other Infections

GOS/FOS supplementation for one year showed a small reduction in the incidence of urinary tract infections during a period of two years in one study [26]. In one trial, there were zero versus four urinary tract infections in a total population of 194 participants after adding HMOs [37]. 

### 4.6. Antibiotic Use

Concerning the addition of GOSs/FOSs to infant formula, three trials showed a reduction in antibiotic use [26,27,30], which is disputed by three other studies [31,32,33] in which there was no difference. The reduction in antibiotic use after adding HMOs to infant formula was only demonstrated in one study [28], while it was refuted in two others [37,38]. Supplementation with GOSs or GOSs/PDX did not influence the incidence of antibiotic use [39,40]. A GOS/PDX trial suggested a reduction in recurrent (>3 times/year) use [39,40].

### 4.7. Variables and Confounders

Some variables and confounders in the different trials could have had an influence on the study outcomes and the infection rates:

#### 4.7.1. Probiotics

The systematic review of Zhao in 2022 concluded that probiotics supplemented to infant formula have a positive effect on URTIs in infants [20]. Considering this, the possible effect of the prebiotic could have been underestimated in the studies where a probiotic was added to infant formula in both the intervention and the control groups, such as the RCTs of Storm et al. and Bocquet et al. [31,35].

#### 4.7.2. Breastfeeding

The literature shows that breastfeeding during the first four to six months has a significant influence on the infection rate during infancy [44,45]. However, some studies already show an advantage after breastfeeding the first two weeks [46]. It is interesting to reflect on the possible (positive) effects of breastfeeding in the included trials. In most of the studies, the influence of breastfeeding could be considered to be low, as the infants were included before the age of 2–4 weeks. In contrast, in the study of Bruzzese et al., it was impossible to exclude a possible effect of breastfeeding; hence, exclusive breastfeeding during the first two weeks of life was an inclusion criterion [27]. Moreover, inclusion was allowed until the age of four months; thus, breastfeeding was possible during this period. The same influence of breastfeeding can be discussed in the other studies. Furthermore, in some studies, breastfeeding was allowed in the intervention group, along with the infant formula containing the prebiotic, without specifying how much breastfeeding was given [26,27,30,32,33].

#### 4.7.3. Dose and Intervention Period

There is an important heterogeneity in the different studies concerning the dose of prebiotics. In the subcategory of GOSs/FOSs, a range of 4 to 8 g/L was added to infant formula in the different trials. This variety was even greater in interventional studies with HMOs, which were supplemented between 0.25 g/L and 5.8 g/L.

If we look at the time period in which the prebiotic is taken, there is a diversity between six weeks and one year. If we take into account that most of the literature suggests that breastfeeding has the greatest advantage against infection during the first six months of life, we could hypothesise that it would be beneficial to add the prebiotic at least during this period. However, it remains unclear what supplemental advantage is offered by adding the prebiotic to infant formula for a longer period.

In the studies in which the observation time was longer than the intervention period, different outcomes were noted. Puccio noted a health advantage at least until the age of 12 months, after taking HMOs until the age of six months [28]. This prolonged prebiotic effect was also noted in the trial of Arslanoglu using scGOSs/lcFOSs [26]. Regarding the intake of GOSs/PDX, they only observed a reduction in respiratory infections during the 48 weeks in which the prebiotic was taken, but this effect was no longer observed in the following 48 weeks [40]. 

#### 4.7.4. Type of Prebiotic

The first studies examined the effects of a combination of short-chain GOSs and long-chain GOSs. The currently available HMOs are all short-chain HMOs. At present, there are no comparative studies examining the differences between short- and long-chain prebiotics. Hence, it is impossible to conclude whether the complexity of the OS has an influence on the prebiotic effect and the infection rate.

#### 4.7.5. Low Infection Rates

The mean annual infection rate in children under three years is three to five [17,18]. In the studies investigated in this review, the incidence of infection in infants and young children varied widely but was lower than expected overall. The low infection rates in the different trials could have led to minimising the observed effect. 

A possible confounder in the number of infections registered is the diagnostic criteria for infection. For example, in the study of Sierra et al., all diagnoses had to be made by a paediatrician. In the study of van Stuijvenberg et al., only a fever of >38.5 °C was a criterion for infection. Both of these examples could have led to an underestimation of the actual incidence. This is in contrast to the inclusion criteria in the study of Bocquet et al., where parents were asked to keep a diary and to note all signs of infections [31]. These diaries were reviewed by a paediatrician on a regular basis, and diagnoses could be made retrospectively. 

Another important confounder could be found in the study of Lasekan et al. The inclusion and follow-up of patients occurred during the COVID-19 pandemic. It is known that the prevalence of all types of infections was very low in children during this period, as a result of quarantine and the limitation of social contacts [47]. 

#### 4.7.6. Atopy/Allergy

In some studies, parental atopic history was an exclusion criterion, focusing the research on healthy infants—for example, in the trials of Van Stuijvenberg et al. and Bocquet et al. [31,32,33]. In contrast, parental history of atopy was an inclusion criterion in other studies [26,30]. Given the fact that parental history of atopy enhances the risk of atopy for the child, and that atopy is relied to more infections [48,49], it might be possible that those children would receive a greater advantage from the prebiotic effect. This assumption has not yet been investigated. 

#### 4.7.7. Caesarean Section

Caesarean section is also considered to be a contributor to enhanced infection rates. Only in the study of Puccio et al. did a sub-analysis show a more important prebiotic effect in the infants born by caesarean than in those born by vaginal birth [28].

#### 4.7.8. Setting

We may hypothesise that with the current high socioeconomic hygienic standards and low infection rates, the supplementation of prebiotics does not trigger a change great enough to detect on a large scale. It could be interesting to investigate the differences in outcomes related to place and socioeconomic setting.

These variables and confounders create an important heterogeneity in the different publications, making it impossible to draw stringent conclusions or conduct meta-analyses. 

### 4.8. Systematic Reviews

No systematic reviews are discussed in this review, as they combine all types of prebiotics in their analyses. However, prebiotics have been reviewed in multiple systematic reviews in different ages. In the systematic review of Skorka et al., published in 2018, the effects of prebiotics in infant formula were explored. In this review, trials with HMOs were excluded. They concluded that supplementation with FOSs, GOSs, and/or acidic OSs had no influence on the frequency of gastrointestinal infections, respiratory tract infections, or antibiotic treatment. Their main conclusion was that there is no existing robust evidence to recommend the routine use of prebiotic-supplemented formula, but they attributed this to a lack of strong evidence [24]. Williams et al., in 2021, published a systematic review on the effects of prebiotics, synbiotics, and short-chain fatty acids on respiratory tract infections and immune function in all ages. This review concluded that supplementation with prebiotics—especially Oss—may play a role in reducing the incidence and duration of respiratory tract infections in infants and children. There is insufficient evidence of this protective effect for the adult population [50]. Rashidi et al. concluded in 2021 that supplementation with prebiotics, probiotics, and synbiotics in infant formula (PRO-formula) plays a protective role against respiratory tract infections. However, analyses of the different subgroups showed no significant association between the consumption of probiotics, prebiotics, or synbiotics and respiratory tract infections [29].

### 4.9. Future

Taking into account the possible effects of the variables and confounders, more comparative research should be initiated to investigate their influence on the outcome.

In addition, in the existing literature, only a limited number of HMOs have been examined. Indeed, 2′Fl and LNnT are the two most common HMOs in human milk. However, it remains uncertain which HMOs provide a protective effect against infections. Hopefully, the ongoing study of van Stigt et al. will give us more clarity on the different components of human milk and their protective role against respiratory tract infections [51].

Furthermore, once the positive effects of the types of prebiotics are more clear, it will be interesting to investigate the cost–benefit analysis of the prebiotics added to infant formula to reduce the incidence of dropout of day care, hospitalisations, medications, parents staying home, etc., taking into account the different costs of the production of the different OSs. To conduct this analysis, more data are needed about the infection duration, which has only been discussed in a few studies. 

## 5. Conclusions

Due to study heterogeneity, it remains impossible to formulate a consensus about the efficacy of adding prebiotics to infant formula. All currently established prebiotics are well tolerated. FOS/GOS supplementation seems to have a beneficial effect on infection rates. For HMOs, more studies about the different types of HMOs are necessary to make any deductions. GOSs, inulin, and MOSs do not reduce the incidence of infections. However, inulin seems to have a positive effect on the duration of infection. The combination of GOSs and PDX showed a protective role in one study. The evidence of the effects of prebiotics in reducing the use of antibiotics is low. 

## Figures and Tables

**Table 1:** Research question and PICO (population, intervention, control, and outcomes) formulation.

Is there a link between the type of prebiotic added to infant formula and the reduction in the incidence of infection in infants?	
Patient	Term healthy infants < 6 months
Intervention	Prebiotics added to infant formula
Control	Infant formula
Outcome	Primary outcome: Reduction in the incidence of infections Secondary outcomes: Reduction in the prescription of antibiotics and their links with the type of prebiotic. Effects on subtypes of infection (e.g., upper airway, lower airway, intestinal infection, others)

**Table 4:** Overview of references with other prebiotics.

First author, Year [Reference]	Country	Inclusion Criteria; Age at Inclusion	Intervention [Number Included]	Control [Number Included]	Duration a. Intervention b. Observation	Outcome
Sierra, 2014 [39]	Spain	Healthy term infants; <8 weeks	Infant formula + GOSs 4.4 g/L Follow-on formula + GOSs 5 g/L (177)	Infant formula Follow-on formula (188)	a. 1 year b. 1 year	Primary outcomes ● Effects on intestinal microbiota: lower faecal pH (*p* = 0.019); lower decreasing trend of secretory IgA (*p* = 0.08); lower butyric acid concentration (*p* = 0.04); ncreased *Bifidobacterium* counts (*p* = 0.01) ● Frequency of defecation higher (*p* < 0.001) ● Softer stools (*p* < 0.05) ● Infections: - Episodes of URTI/infant: 1.84 ± 2.01 vs. 1.65 ± 1.83 (*p* = 0.4) - Episodes of diarrhoea/infant: 0.27 ± 0.67 vs. 0.20 ± 0.52 (*p* = 0.36) - Use of antibiotics: 17.8% vs. 19.8% (*p* = 0.48) ● Allergic manifestations: 39/132 vs. 28/132 (*p* = 0.12)
Ranucci, 2018 [40]	Italy	Term infants with parental atopic history; Day 0	Infant formula + GOSs/PDX 1:1 4 g/L (+/− BF) (201)	● Infant formula (+/− BF) (199) ● Breastfeeding (140)	a. 48 weeks b. 96 weeks	Primary outcome ● Incidence of atopic dermatitis: - at 48 weeks: 49/118 vs. 50/104 (*p* = 0.62) - at 96 weeks: 56/118 vs. 60/104 (*p* = 0.28) Secondary outcomes ● Infections - Infants with at least 1 RTI at 48 weeks: 39/118 vs. 50/104 (*p* = 0.023) at 96 weeks: 77/118 vs. 73/104 (*p* > 0.05) - Patients with RRI until 96 weeks: 24/118 vs. 33/104 (*p* = 0.039) - Antibiotics prescribed: 58/118 vs. 58/104 (*p* > 0.05) - Patients > 3 x antibiotics prescribed: 22/118 vs. 26/104 (*p* = 0.056) - Acute gastroenteritis: 62/118 vs. 65/104 (*p* = 0.064) ● Intestinal microbiology: - *Bifidobacteria* load higher (*p* = 0.01) - Link between RI and lower *Bifidobacteria* load
Nomayo, 2020 [33]	Germany	Healthy term infants; <10 days	Infant formula+ high amounts β-PA (20–25%) + GOS 5 g/L (47)	● Infant formula (β-PA <10%) (47) ● Breastfeeding (34)	a. 12 weeks to 6 months b. 1 year	Primary outcome s ● Faecal *Bifidobacteria*: total count median 4.4 ± 5.4 × 108 vs. 0.7 ± 2.1 × 108 (*p* < 0.01) ● Infections: Overall: 3/22 vs. 2/19 (*p* = 0.65) - GI infections: 0/22 vs. 0/19 - RTI: 3/22 vs. 2/19 (*p* = 0.65)
Estorninos, 2021 [35]	Philippines	Healthy term infants; 21—26 days	Alpha-lactalbumin and sn-2 palmitates enriched infant formula + MOSs 7.2 g/L (115)	Alpha-lactalbumin and sn-2 palmitates enriched infant formula (115)	a. 6 months b. 6 months	Primary outcome ● Weight gain similar (*p* = 0.7) ● Stool consistency was softer (*p* = 0.005) ● Tolerance: similar Secondary outcomes ● Adverse events: - URTIs: 59/115 vs. 57/115 - LRTIs: 24/115 vs. 24/115
Neumer, 2021 [36]	Spain, Belgium	Healthy term infants; <4 months	Infant formula + sc/lc inulin 1:1 8 g/L (81)	Infant formula (79)	a. 1 year b. 1 year	Primary outcomes ● Number of infections: mean 0.64 ± 1.05 vs. 0.55 ± 1.04 (*p* > 0.05) ● Lower mean duration infections in intervention group (*p* = 0.034) Secondary outcomes ● Anthropometric measures: similar ● Allergic manifestations: overall very low ● Wellbeing: total daily crying lower, but NS (*p* = 0.06) ● Gastrointestinal tolerance: similar ● Intestinal microbiota: - Total bacterial counts were similar; - *Bifidobacterium* count at 6 months: median 8.91 [8.31–9.41] vs. 8.15 [7.24–9.09] (*p* = 0.06); difference smaller at 12 months (*p* = 0.5)

Results are written as absolute values or as mean values ± standard deviations. BF: breastfeeding, GOS: galactooligosaccharide, FOS: fructooligosaccharide, β-PA: beta palmitic acid, MOS: bovine-milk-derived oligosaccharide, PDX: polydextrose, (L)RTI: (lower) respiratory tract infection, lc: long-chain, sc: short-chain, GI: gastrointestinal; NS: not significnat.

## Data Availability

Not applicable.
